# The Effect of Rhythmic Audio-Visual Stimulation on Inhibitory Control: An ERP Study

**DOI:** 10.3390/brainsci14050506

**Published:** 2024-05-17

**Authors:** Yifan Wang, Di Wu, Kewei Sun, Yan Zhu, Xianglong Chen, Wei Xiao

**Affiliations:** Department of Military Medical Psychology, Air Force Medical University, Xi’an 710032, China; wyyff_1220@163.com (Y.W.); wudi0426@outlook.com (D.W.); xlxsunkewei@126.com (K.S.); 13898277961@163.com (Y.Z.); qq17687658@163.com (X.C.)

**Keywords:** inhibitory control, response inhibition, conflict inhibition, rhythmic audio-visual stimuli, Go/NoGo task, Stroop task, ERP, N2

## Abstract

Inhibitory control, as an essential cognitive ability, affects the development of higher cognitive functions. Rhythmic perceptual stimulation has been used to improve cognitive abilities. It is unclear, however, whether it can be used to improve inhibitory control. This study used the Go/NoGo task and the Stroop task to assess various levels of inhibitory control using rhythmic audio-visual stimuli as the stimulus mode. Sixty subjects were randomly divided into three groups to receive 6 Hz, 10 Hz, and white noise stimulation for 30 min. Two tasks were completed by each subject both before and after the stimulus. Before and after the task, closed-eye resting EEG data were collected. The results showed no differences in behavioral and EEG measures of the Go/NoGo task among the three groups. While both 6 Hz and 10 Hz audio-visual stimulation reduced the conflict effect in the Stroop task, only 6 Hz audio-visual stimulation improved the amplitude of the N2 component and decreased the conflict score. Although rhythmic audio-visual stimulation did not enhance response inhibition, it improved conflict inhibition.

## 1. Introduction

Executive function refers to the general control mechanism that coordinates various cognitive processes and behaviors to ensure the realization of specific cognitive goals when completing complex tasks, including inhibitory control, working memory, and cognitive flexibility [1,2]. Inhibitory control and working memory support each other, and cognitive flexibility is based on inhibitory control and working memory [3]. 

Inhibitory control refers to an individual’s ability to control their thoughts, emotions, or behaviors in order to overcome a strong dominant response or external interference in order to achieve a desired goal [3,4]. Inhibitory control, as a basic cognitive ability, is closely related to higher cognitive functions. For example, inhibitory control can inhibit false concepts and make unique contributions to scientific reasoning [5,6], and the improvement in inhibitory control ability can also improve decision-making [7,8]. Inhibitory control is closely related to fluid intelligence, and enhancing inhibitory control can improve fluid intelligence [9,10]. Depression [11,12], obsessive–compulsive disorder [13,14], obesity [15,16], addictive habits [17,18], etc., are also closely related to impaired inhibitory control. In conclusion, inhibitory control, as an essential cognitive function, is particularly important for individual healthy development.

Inhibitory control is divided into response inhibition and conflict inhibition. The capacity to suppress a dominant response or abandon a goal when it is no longer necessary or when it is possibly dangerous is known as response inhibition [19,20]. Additionally, the ability of the brain to detect and settle conflicts between information when it arises and gets in the way of processing valid information and accomplishing a goal is known as conflict inhibition [21]. The most common method for measuring response inhibition is the Go/NoGo task and the Stop Signal task [22,23]. Conflict inhibition is typically assessed using the Stroop task [24].

ERPs are minute voltages that the brain produces in reaction to a particular stimulus or event [25,26]. ERPs are often detected by EEG and are evaluated based on their amplitude [27,28]. The primary event-related potential (ERP) connected to inhibitory control is N2 (negative 2) [29,30], which typically manifests 200–350 ms after the stimulus [31,32]. The N2 component is indicative of visual attention processing and cognitive control, which includes error monitoring, response inhibition, response conflict, and conflict monitoring [33,34,35]. A greater N2 wave amplitude is indicative of stronger inhibitory control ability, according to prior studies [36,37,38]. 

Inhibitory control is pliable and scalable. Prior research has mostly employed three methods to improve cognitive function: cognitive behavioral training, positive thinking and exercise training, and noninvasive brain stimulation techniques [38,39,40,41]. Noninvasive brain stimulation primarily refers to the introduction of electrical, magnetic, auditory, and visual stimuli to the brain to modify neural activity and enhance cognitive function [42,43]. Noninvasive burrs are faster and more effective than the other two approaches, which take considerable time.

Transcranial alternating current stimulation (tACS), transcranial magnetic stimulation (TMS), and rhythmic perceptual stimulation (e.g., fixed-frequency sound or picture stimulation) in noninvasive brain stimulation are also known as rhythmic stimuli. The regulation of cognitive function by rhythmic stimulation is based on brain entrainment. External rhythmic stimulation is applied to the brain, which causes changes in neural oscillations in the brain and thus affects cognitive function [43]. It was found that 6 Hz tACS reduced the conflict effect in the Stroop task and regulated the conflict processing process [44]. Stacey found that tACS at 10 Hz could be an effective means of modulating response inhibition compared to gamma frequency stimulation [45]. Moreover, high-frequency repetitive transcranial magnetic stimulation (HF-rTMS) at 10 Hz not only improved inhibitory control in healthy young adults but also improved attention control in aging individuals [46,47,48]. Therefore, 6 Hz and 10 Hz are effective stimulation frequencies and will also be used in this study.

Rhythmic perceptual stimulation is a form of rhythmic stimulation that refers to perceptual stimuli that are presented at a fixed frequency, including fixed-frequency visual brightness changes, visual flicker stimulation, and audio-visual synchronization stimulation [49,50]. Rhythmic perceptual stimulation shares the same mechanism of action as the above two techniques and has the advantages of being highly manipulable, low-cost, and not inducing additional neural activity [51]. Rhythmic perceptual stimulation has been found to be useful for preoperative and postoperative mood regulation [52,53], as well as for the rehabilitation of Parkinson’s disease patients [54,55] and stroke patients [56]. Rhythmic perceptual stimulation has also been used to improve cognitive abilities such as attention, working memory, situational memory, cognitive flexibility, divergent thinking, and creativity [57,58,59,60,61]. It has been shown that 40 Hz audio-visual stimulation can modulate brain region function and it has been used to improve cognitive impairment, movement disorders, mood, and sleep disorders [62]. According to Brooke, memory extraction was made easier after 30 min of theta-band rhythmic audio-visual stimulation [57]. The aforementioned studies have demonstrated that rhythmic audio-visual stimulation is a safe and dependable method of neuromodulation. 

However, rhythmic perceptual stimuli have been used to regulate some cognitive abilities, but it is unclear whether they can be used to regulate inhibitory control. Therefore, in this study, the Go/NoGo task and the Stroop task were used to measure response inhibition and conflict inhibition, and rhythmical audio-visual stimulation was used as the stimulus mode. This study will explore the regulatory effects of rhythmic audio-visual stimuli on inhibitory control from the perspective of behavioral and ERPs, in order to provide a new method for strengthening inhibitory control.

## 2. Materials and Methods

### 2.1. Experimental Protocol

**Figure 1:**
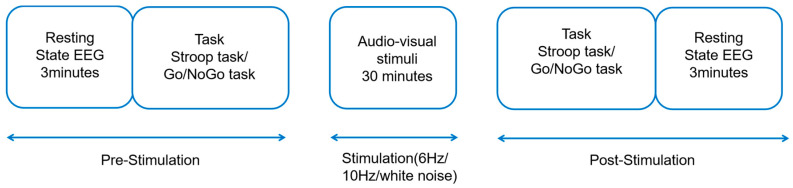
Experimental design. Rhythmic audio-visual stimulation lasted 30 min. Stroop task and Go/NoGo task were executed before and after experiment, and EEG data were collected throughout experiment.

In this study, a single-blind, false-controlled experimental design was adopted, and the experiment was carried out in a laboratory with sound insulation, weak lighting, and electromagnetic shielding. In the practice phase, the main subject explained the experimental procedure in detail, and the subjects read the task instructions, in which the Go/NoGo task and the Stroop task were practiced for 15 trials. In the formal experimental phase, the first phase, the participants provided a 3 min EEG resting state measurement and then completed the baseline measurement of the two tasks. In the second stage, throughout the thirty minutes of audio-visual stimulation, the subjects were instructed to wear headphones to stay awake and focus on the screen. In the third stage, after the rhythmic audio-visual stimulation, participants performed two tasks and received 3-min EEG resting state measurements. The experimental process is shown in Figure 1.

### 2.2. Participants

We advertised posters on the WeChat platform and recruited 60 students (30 males and 30 females; mean age of 22 ± 1.54 years) in a military medical school. Three groups of individuals were randomly assigned: one for white noise, one for 10 Hz stimulation, and one for 6 Hz stimulation. Through questionnaires and self-reports, we ensured that all subjects were right-handed, had normal vision and hearing, had no music learning experience, and had not participated in similar experiments within six months. We excluded the subjects with psychological problems and alcohol dependence through the periodic psychological test in the school. The day before the experiment, the participants were told to have enough sleep and were forbidden to drink coffee or alcohol. This study was approved by the Medical Ethics Committee of the Air Force Military Medical University. All subjects signed an informed consent form before the experiment, and they were paid after the experiment.

### 2.3. Stimulation Materials

The PsychToolbox in MATLAB 2021b generated both visual and auditory stimuli, which were employed as the stimulus materials in this investigation. The visual stimulus material was a 30 × 30 cm flashing checkerboard grid with 6 Hz, 10 Hz, and random-frequency flashes. Visual stimuli were presented on a 37 × 30 cm computer monitor with 1920 × 1080 spatial pixels and a 59.94 Hz refresh rate. The auditory stimuli were 6 Hz and 10 Hz pure tones and white noise, presented by Huawei AM115 headphones. Visual and auditory stimuli were presented synchronously in the experiment. The screen brightness was 60 cd/m^2^ and the sound intensity was 60 dB [59]. The subjects were 70 cm from the screen. The subjects could adjust their position for comfort.

### 2.4. Experimental Task

#### 2.4.1. Go/NoGo Task

**Figure 2:**
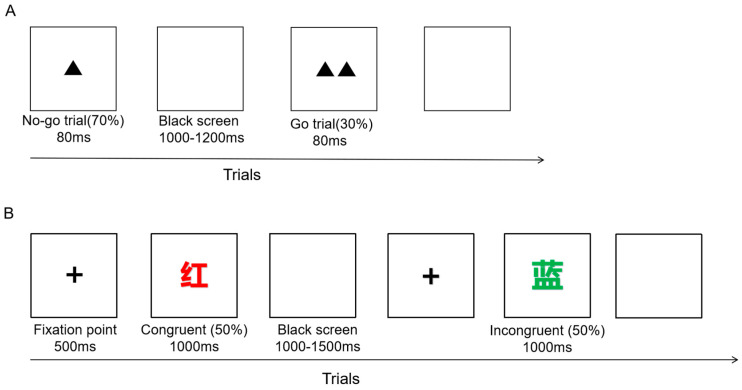
Task procedure. (**A**) Go/NoGo task; (**B**) Stroop task.

The Go/NoGo task was presented by the E-prime 3.0 software on the computer. There were 400 trials, divided into 2 blocks, and each block had 200 trials. Subjects in each block were given a break and could press any key to continue the task when they were ready. The Go/NoGo task was categorized into single- and double-triangular stimuli. The single triangle represented the go signal, appearing in 280 trials, and the double triangle represented the no-go signal, appearing in 120 trials. The two stimuli were presented randomly in the center of the screen for 80 ms each, and the stimulus intervals were randomly varied from 1000 ms to 1200 ms. The subjects were required to press the keyboard “←” when the single-triangular stimulus was presented and to suppress the keypress impulse and not respond when the double-triangular stimulus was presented. The task flow chart is shown in Figure 2A. 

#### 2.4.2. Stroop Task

The Stroop task was presented by E-prime 3.0 software on the computer with a total of 240 trials. The task stimulus contains four Chinese characters (“red”, “yellow”, “green”, and “blue”). Each Chinese character was randomly presented in one of the four colors (red, yellow, green, and blue). When the meaning of the word matched the font color, it was a congruent condition, and there were 120 such trials. When the meaning of the word was inconsistent with the color of the font, it was an incongruent condition. There were 120 such trials. Participants needed to ignore the meaning of the word and respond according to the color of the word by pressing certain buttons on a keyboard. Red, blue, green, and yellow corresponded to the F, G, J, and K buttons on the keyboard, respectively. In the task, a fixed point was presented first for 500 ms, followed by the presentation of a Chinese character for 1000 ms. The subjects were given 1800 ms to react. And then, a blank screen was presented for 1000–1500 ms. (Figure 2B). 

### 2.5. EEG Recording

The 32-electrode BrainAmp EEG system manufactured by the Brain Products Company was utilized in this investigation to record EEG signals in accordance with the 10–20 system electrode locations. The online bandpass filter was set to 0.01–100 Hz, the grounding electrode was labeled GND, the online reference electrode was Fz, the impedance between the scalp and electrode was less than 10 kΩ, and the EEG equipment had a sampling rate of 1000 Hz. During the EEG recording period, the subjects were asked to minimize their head and eye movements, to focus on concentrating, and to maintain an awake state. 

### 2.6. Data Analysis

#### 2.6.1. Behavioral Data Analysis

We calculated the go-trials reaction time and no-go-trials correct rejection rate as behavioral indicators of the Go/Nogo task. Conflict effect and conflict score was the behavioral indicators of the Stroop task. The smaller the response time of the go trials and the higher the correct rejection rate of the no-go trials, the stronger the response inhibition ability. The smaller the conflict effect or conflict score, the better the interference inhibition ability.

We utilized SPSS 23.0 to conduct K-S tests on the data, and all of the results were *p* > 0.05. The data of all participants were normally distributed. Finally, a 2 (time: pre-test and post-test) × 3 (stimulus type: 6 Hz, 10 Hz, and white noise) repeated-measures ANOVA was performed on each of the three metrics in the experiment to assess the effect of rhythmic audio-visual stimuli on inhibitory control. The Bonferroni test was used for multiple comparison correction. The test level was *α* < 0.05. Marginal significance was considered when 0.05 < *p* < 0.1 [63]. All of the statistical analyses were performed using SPSS 23.0 software.

#### 2.6.2. EEG Data Analysis

The preprocessing of offline data was performed using MATLAB 2021b and the EEGLAB toolbox. Channel localization was followed by a 48–52 Hz notch filter and a 0.1–40 Hz bandpass filter. The bilateral mastoids were used as the reference electrodes. The EEG data from the Stroop task were segmented from 200 ms pre-stimulation to 1000 ms post-stimulation. The EEG data from the Go/NoGo task were segmented from 200 ms pre-stimulation to 800 ms post-stimulation. The baseline correction was 200 ms. We checked the segmented waveforms and deleted the clips with messy and fluttering waveforms. If a channel waveform is always in an irregular state, it is recognized as a bad guide and needs to be calculated through interpolation. The terrain interpolation method was used to calculate the bad channel values, and the data pertaining to more than three problematic channels were omitted [64]. Electro-oculogram and electromyogram artifacts were eliminated by independent component analysis (ICA) [65]. After ICA processing, we observed whether there were ocular and myoelectric artifacts in the fragments again, so as to ensure the successful elimination of artifacts. Then, the artifacts with amplitudes greater than 100 μV or less than −100 μV were discarded. 

In this study, all trials of the Stroop task were categorized by congruent and incongruent conditions, and the ERP data of the incongruent condition were superimposed and averaged. All trials of the Go/NoGo task were categorized by the go stimulus and the no-go stimulus, and the ERP data of the no-go stimulus were superimposed and averaged. The observation period ranged from −200 ms to 800 ms. By observing the waveform graphs of the groups, we determined that the time window of the N2 component in the Stroop task was 200–350 ms and that of the N2 component in the Go/NoGO task was 200–300 ms. The prefrontal lobe is the representative of inhibitory control, meaning inhibitory control, as a top-down mechanism, is activated by the prefrontal lobe to inhibit the activity of the effect or sensory system [66,67]. Therefore, F3, Fz, and F4 frontal lobe electrodes were selected for analysis. 

A two-factor repeated-measures ANOVA [2 (time: before stimulus and after stimulus) × 3 (stimulus type: 6 Hz, 10 Hz, and white noise)] was used to calculate the N2 amplitude. The Bonferroni test was used for multiple comparison correction. The test level was *α* < 0.05. When 0.05 < *p* < 0.1, it was considered marginally significant. 

## 3. Results

We conducted a one-way ANOVA on the behavioral measures of the Stroop task and the Go/NoGo task using different stimulus groups (6 Hz, 10 Hz, and white noise) as between-subject factors. The results showed that there was no difference in the baseline performance of conflict effects and conflict scores among the three groups of subjects (*F*_(2,57)_ = 0.021, partial *η*^2^ = 0.001, and *p* = 0.979; *F*_(2,57)_ = 0.457, partial *η*^2^ = 0.016, and *p* = 0.636) in the Stroop task. There was also no significant difference in the baseline performance of go-trial response times and no-go-trial correct rejection rate (*F*_(2,57)_ = 0.155, partial *η*^2^ = 0.005, and *p* = 0.857; *F*_(2,57)_ = 0.743, partial *η*^2^ = 0.025, and *p* = 0.480) in the Go/NoGo task. Therefore, the randomization was successful. Partial *η*^2^ = 0.04 indicates a small effect size, partial *η*^2^ = 0.25 indicates a medium effect size, and partial *η*^2^ = 0.64 indicates a large effect size.

### 3.1. Stroop Task Behavioral Results

**Figure 3:**
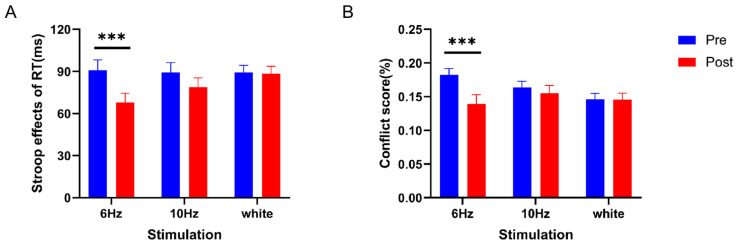
In the Stroop task, means and standard errors for Stroop effects and conflict scores were calculated. (**A**) Stroop effects in the 6 Hz, 10 Hz, and white noise groups are shown. (**B**) Conflict scores in the 6 Hz, 10 Hz, and white noise groups are shown. The error bars represent the standard error; *p* < 0.001 ***.

In the statistical analysis of the effect of audio-visual stimulation on the Stroop task, we performed a repeated-measures ANOVA on conflict effects and conflict scores by using different stimulus groups (6 Hz, 10 Hz, and white noise) as between-subject factors and before and after stimulation, i.e., time, as a within-subject factors. The ANOVA results for the conflict effect showed a significant main effect of time (*F*_(1,57)_ = 12.061, partial *η*^2^ = 0.175, and *p* < 0.01), a nonsignificant main effect of stimulus group (*F*_(2,57)_ = 0.652, partial *η*^2^ = 0.022, and *p* > 0.05), and a significant interaction effect (*F*_(2,57)_ = 3.763, partial *η*^2^ = 0.117, and *p* < 0.05). Simple effects analysis showed that compared with the white noise group, the conflict effect of 6 Hz and 10 Hz was reduced (*p* < 0.001; *p =* 0.072) (See Figure 3A). 

The ANOVA results of the conflict scores showed a significant time main effect (*F*_(1,57)_ = 7.129, partial *η*^2^ = 0.111, and *p* < 0.05). The stimulus main effect was not significant (*F*_(1,57)_ = 0.849, partial *η*^2^ = 0.029, and *p* > 0.05), but the interaction effect was significant (*F*_(1,57)_ = 3.947, partial *η*^2^ = 0.122, and *p* < 0.05). Simple effects analysis showed that conflict scores were significantly lower in the 6 Hz group (*p* < 0.001), and the conflict scores in the 10 Hz and white noise groups were not significantly different (see Figure 3B).

### 3.2. Go/NoGo Task Behavioral Results

**Figure 4:**
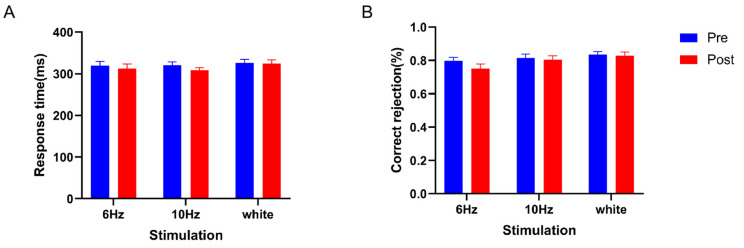
In the Go/NoGo task, the response time of the go trials and the correct rejection rate for the no-go trials were calculated. (**A**) The go-trial response times in the 6 Hz, 10 Hz, and white noise groups are shown. (**B**) For the no-go trials, the correct rejection rate in the 6 Hz, 10 Hz, and white noise groups is shown. The error bars represent the standard error.

The repeated-measures ANOVA of the go-trial response times showed a significant main effect of time (*F*_(1,57)_ = 4.305, partial *η*^2^ = 0.070, and *p* < 0.05), a nonsignificant main effect of stimulus type (*F_(_*_2,57)_ = 0.462, partial *η*^2^ = 0.016, and *p* > 0.05), and a nonsignificant interaction effect (*F*_(2,57)_ = 0.672, partial *η*^2^ = 0.032, and *p* > 0.05) (See Figure 4A). The results indicate that different stimulus types had no effect on the go-trial response times. 

The repeated-measures ANOVA for the no-go-trial correct rejection rate revealed a significant time main effect (*F*_(1,57)_ = 4.611, partial *η*^2^ = 0.075, and *p* < 0.05), a nonsignificant stimulus main effect (*F*_(2,57)_ = 1.701, partial *η*^2^ = 0.056, and *p* > 0.05), and a nonsignificant interaction effect (*F*_(2,57)_ = 1.550, partial *η*^2^ = 0.052, and *p* > 0.05) (See Figure 4B). The results show that different stimulus types had no effect on the no-go-trial correct rejection rate. 

In summary, different stimulation conditions did not improve the behavior of response inhibition.

### 3.3. ERP Results

#### 3.3.1. Effects of Audio-Visual Stimulation on N2 during Stroop Task

**Figure 5:**
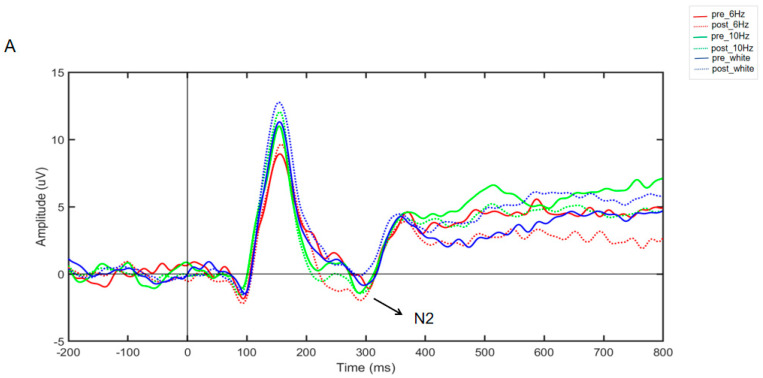

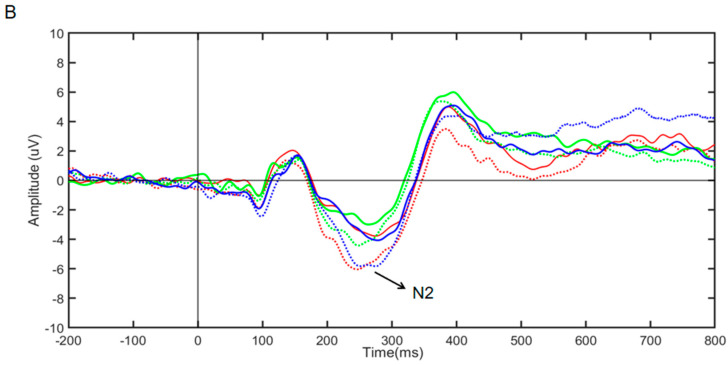
(**A**) Average ERP waveform in Stroop task. (**B**) Average ERP waveform in Go/NoGo task. Note: pre_6 Hz, pre_10 Hz, and pre_white represent baseline measurements of task before stimulation; post_6 Hz, post_10 Hz, and post_white represent task measurements after stimulation.

According to Figure 5A, the repeated-measures ANOVA results for the N2 component amplitude for the three groups of subjects showed a nonsignificant time main effect (*F*_(1,57)_ = 1.809, partial *η*^2^ = 0.013, and *p* > 0.05), a nonsignificant stimulus main effect (*F*_(2,57)_ = 0.214, partial *η*^2^ = 0.013, and *p* > 0.05), and a significant interaction effect (*F*_(2,57)_ = 3.508, partial *η*^2^ = 0.808, and *p* < 0.01). Simple effects analysis revealed that the N2 component amplitude was significantly greater in the 6 Hz group (*F*_(1,57)_ = 6.256, partial *η*^2^ = 0.099, and *p* < 0.05); there was no significant difference in the N2 component amplitude between the 10 Hz group and the white noise group [(*F*_(1,57)_ = 1.091, partial *η*^2^ = 0.019, and *p* > 0.05); (*F*_(1,57)_ = 1.478, partial *η*^2^ = 0.025, and *p >* 0.05)]. The results suggest that only rhythmic audio-visual stimulation at 6 Hz enhanced conflict inhibition, while the 10 Hz rhythmic audio-visual stimulus and white noise groups had no effect.

#### 3.3.2. The Effects of Audio-Visual Stimulation on the N2 Component of the Go/NoGo Task

As shown in Figure 5B, the repeated-measures ANOVA results for the N2 component amplitude for the three groups of subjects showed a significant time main effect (*F*_(1,57)_ = 27.837, partial *η*^2^ = 0.328, and *p* < 0.001), a nonsignificant stimulus main effect (*F*_(2,57)_ = 0.626, partial *η*^2^ = 0.021, and *p* > 0.05), and a nonsignificant interaction effect (*F*_(2,57)_ = 0.853, partial *η*^2^ = 0.029, and *p* > 0.05). This showed that no matter the type of stimulus, the amplitude of the N2 component will be affected by the time factor, that is, the change in the N2 component amplitude is the result of the practice effect. Rhythmic audio-visual stimuli had no effect on the amplitude of the N2 component of the Go/NoGo task.

## 4. Discussion

The present study examined the modulation of inhibitory control via rhythmic audio-visual stimuli in the Stroop and Go/NoGo tasks. Both 6 Hz and 10 Hz rhythmic audio-visual stimuli (compared with white noise) improved conflict inhibition behavioral performance, but no matter the kind of stimulus, it did not improve response inhibition behavioral performance. Furthermore, only the 6 Hz rhythmic audio-visual stimuli enhanced the amplitude of the N2 component in the Stroop task, while the enhancement of the N2 amplitude represented an improvement in conflict inhibition ability. The 6 Hz rhythmic audio-visual stimulation effectively improved conflict inhibition ability.

Our results reveal that subjects receiving 6 Hz and 10 Hz rhythmic audio-visual stimuli reacted faster in the conflict condition compared to subjects receiving white noise stimuli; the conflict effect was reduced, and the 6 Hz rhythmic audio-visual stimuli also reduced the conflict score. Previous studies have shown that 10 Hz rTMS reduces the conflict effect reaction time [47], and 6 Hz tACS is able to reduce conflict effects and conflict scores in the Stroop task [44,68]. Zhu et al. [46] found that tACS at 6 Hz and 10 Hz significantly improves behavioral performance in the Stroop task. These studies are consistent with our findings.

Respectively, 6 Hz and 10 Hz belong to the theta and alpha bands. Previous studies have found that successful control produces larger theta oscillations during cognitive control tasks [69,70]. Theta oscillations in the frontal midline are a mechanism of cognitive control, and their increase represents an increase in cognitive control [71,72]. Theta oscillation is also associated with conflict processes and is closely related to conflict detection and resolution capabilities [73,74]. Alpha oscillation is a gating mechanism of inhibition which can suppress irrelevant information, improve cognitive processing efficiency, and promote the success of goal-oriented behavior [75,76,77]. In addition, the increase in alpha oscillations enhances perceptual discrimination [78,79], attention [80], and other cognitive functions. Moreover, improvements in these abilities also enhance the ability to recognize and monitor conflicting stimuli. In our study, we used rhythmic audio-visual stimulation, which works in the same way as transcranial magnetic or transcranial alternating current stimulation, by introducing exogenous alpha- and theta-band stimulation, which affects neural oscillations within the brain and thus improves the behavioral performance of conflict inhibition. These results suggest that 6 Hz and 10 Hz rhythmic audio-visual stimulation effectively enhance conflict inhibition. 

From the results of ERP analysis, it can be concluded that 6 Hz rhythmic audio-visual stimulation enhanced the amplitude of the N2 component, while 10 Hz rhythmic stimulation and white noise stimulation had no enhancement effect. The N2 component is believed to be related to the mechanism of conflict inhibition and is an important indicator of conflict inhibition, which is closely related to conflict monitoring and conflict resolution in the inhibition process [32,81]. A greater amplitude of the N2 component represents stronger conflict monitoring and resolution ability. For example, Xu found that N2 amplitude increased in subjects who were trained in inhibitory control when they completed the Stroop task compared to subjects who were not trained in inhibitory control [38]. A study of obsessive–compulsive disorder (OCD) patients revealed that compared to healthy subjects, OCD patients had impaired inhibitory control in conflict conditions, as evidenced by the diminished N2 wave amplitude in the Stroop task [82]. Our study revealed a significant increase in the amplitude of the N2 component after subjects received 6 Hz rhythmic audio-visual stimulation, suggesting that 6 Hz audio-visual stimulation enhances subjects’ ability to monitor and resolve conflicting stimuli and improves conflict inhibition. 

Although 10 Hz and 6 Hz improved behavioral performance in conflict inhibition, only rhythmic audio-visual stimulation at 6 Hz increased the amplitude of the N2 component. According to Basar’s empirically based theory of neural oscillatory ensembles, the real activity of the brain is neural oscillation, which reflects the processing of information and is a valid indicator of cognitive processing, and event-related potentials (ERPs) are composite waveforms composed of oscillatory responses superimposed on the characteristics of the responses in a given task condition [83,84]. Theta oscillation is associated with negative ERPs evoked during the performance of a monitoring task [85], such as the N2 component. And theta oscillation contributes most to its waveform amplitude during the latency of the N2 [86]. This may be the reason why 6 Hz rhythmic audio-visual stimulation enhanced the wave amplitude of the N2 component, whereas 10 Hz audio-visual stimulation did not.

Nevertheless, studies have shown that 6 Hz and 10 Hz are effective stimulation frequencies to improve response inhibition. Theta-band tACS stimulation has been shown to be a cognitive enhancement tool that improves cognitive function in healthy older and younger adults [87]. tACS at 10 Hz can improve behavioral performance on the Go/NoGo task [45]. rTMS at 10 Hz has facilitated response inhibition during a stop-signal task in patients with major depression [88].

However, in our study, we found that different types of stimulation did not enhance response inhibition. The possible reasons are as follows. First, the stimulus frequency band employed may not be consistent with task-evoked oscillation. Response inhibition, which is also a motor inhibitory control, is initiated by the right inferior frontal gyrus and mainly involves the modulation of activity in the beta band (13–30 Hz) [89]. Beta power better predicts stop-signal responses when frontal–central beta oscillations are a sign of successful inhibition of movement [90]. Therefore, the rhythmic audio-visual stimuli in the theta and alpha frequency bands that we used may not be suitable for modulating response inhibition. Second, theta- and alpha-band stimuli may impair response inhibition. Although studies have suggested that theta and alpha band share and transmit information to regulate conflict during response inhibition [91], not only is response inhibition involved during a task, other processes, such as error monitoring and sustained attention, are also associated with consistently reduced theta activity in the medial–frontal cortex [92,93]. Studies with anxious individuals have shown that highly anxious individuals exhibit impaired response inhibition and greater alpha activity during response control task performance [94]. Thus, in the present study, we augmented the corresponding neural oscillations within the brain through rhythmic audio-visual stimulation in the theta and alpha frequency bands, which instead interfered with subject performance on a response inhibition task. The third factor is task difficulty. The stimulus effect depends on task difficulty [95]. The Go/NoGo task in this study was a relatively simple key response task that is prone to ceiling effects, which can mask the effect of stimuli on task performance. 

Therefore, we need to use a pre-experiment to assess the difficulty of the task, and we need to increase the difficulty of the task, such as by shortening the stimulus presentation time and changing the stimulus form, so as to better investigate the effects of rhythmic audio-visual stimuli on response inhibition. 

In addition, white noise was used as the control condition in this study. This is because white noise is a reference of neutral sound, it can be used as a fake stimulus group, and the density of the white noise power spectrum is stable and does not depend on the frequency [96,97]. White noise is an effective control condition that does not affect cognitive performance [58,98]. However, studies have found that persistently high levels of white noise can impair higher cognitive functions [99,100]. Alexander found that white noise impaired the ability to resolve conflict, and suggested that the effects of white noise on cognition depend on the task, subject, and stimulus intensity [101]. To sum up, the effects of white noise on cognition need to be further explored, and the effects of white noise on inhibitory control will be further discussed in future studies.

## 5. Conclusions

In conclusion, our study revealed that theta-band and alpha-band rhythmic audio-visual stimuli did not improve response inhibition but were able to enhance conflict inhibition. Rhythmic audio-visual stimulation can effectively modulate conflict inhibition and provide a new approach for future training in conflict inhibition. 

The limitations of this study must also be acknowledged. The subjects in this study were a healthy group of college students. It is not suitable for promotion because of age and health conditions. Future studies may explore the regulatory effects of rhythmic audio-visual stimuli on adolescents with underdeveloped inhibitory control and mental disorders closely related to inhibitory control deficits. Current research results on the effects of white noise on cognition has been contradictory. We will further explore the effects of white noise on inhibitory control by adding the silent group in future studies. In addition, in future studies, we can also add other frequencies such as beta (13–30 Hz) frequency to again explore the regulatory role of rhythmic audio-visual stimuli on response inhibition. We should examine whether multiple stimuli over a long period can enhance the modulation effect and how long the after-effect lasts to provide a better program for conflict inhibition training in the future.

## Data Availability

The data for this article are not available at this time because they are involved in research projects that have not yet been completed. If necessary, requests for access to the dataset should be made by contacting the corresponding author.
